# Editorial: New Insights Into Understanding and Managing NAFLD

**DOI:** 10.3389/fmed.2021.777740

**Published:** 2021-11-12

**Authors:** Óscar Escribano, Daniel E. Francés, Yolanda F. Otero, Javier Egea, Águeda González-Rodríguez

**Affiliations:** ^1^Laboratory of Hepatic and Cardiovascular Diseases, Biochemistry and Molecular Biology Department, School of Pharmacy, Complutense University of Madrid, Madrid, Spain; ^2^Instituto de Fisiología Experimental (IFISE-CONICET), Rosario, Argentina; ^3^Valbiotis, Riom, France; ^4^Molecular Neuroinflammation and Neuronal Plasticity Research Laboratory, Research Unit, Hospital Universitario Santa Cristina, Instituto de Investigación Sanitaria-Hospital Universitario de la Princesa, Madrid, Spain; ^5^Metabolic Syndrome and Vascular Risk Research Laboratory, Research Unit, Hospital Universitario Santa Cristina, Instituto de Investigación Sanitaria-Hospital Universitario de la Princesa, Madrid, Spain; ^6^Centro de Investigación Biomédica en Red de Diabetes y Enfermedades Metabólicas Asociadas (CIBERDEM), Instituto de Salud Carlos III, Madrid, Spain

**Keywords:** NAFLD (non-alcoholic fatty liver disease), biomarkers, therapeutical approaches, liver pathophysiology, steatohepatitis

Non-alcoholic fatty liver disease (NAFLD) is a multisystem disease with complications related to the metabolic syndrome and with a diverse histopathological spectrum ranging from simple steatosis, also termed fatty liver (NAFL) without significant inflammation to steatohepatitis (NASH) with varying stages of fibrosis and, ultimately, cirrhosis, and hepatocellular carcinoma.

Since NAFLD is the most common chronic liver disease worldwide, causing a considerable health burden, there is an increasing number of research groups that are deeply interested in the identification of risk factors involved in the progression of the liver damage, the characterization of new biomarkers with utility for its non-invasive diagnosis and the recognition of novel molecular targets for its treatment.

This special issue contains 18 papers, including 10 original research articles and 8 reviews, reporting important data about novel perspectives regarding pathogenesis and management of NAFLD.

Although NAFLD has been conceived as a different entity from alcohol-related fatty liver disease (ALD), both diseases have an overlap in the pathophysiology, share genetic-epigenetic factors, and frequently coexist. In this sense, Idalsoaga et al. reviewed the overlapping pathophysiology of NAFLD and ALD, and also the effects of metabolic dysfunction and overweight in ALD (Idalsoaga et al.).

The identification of risk factors involved in NAFLD progression is important to develop preventive interventions. In this context, Charatcharoenwitthaya et al. described a detrimental effect of cigarette smoking on all-cause mortality in the study cohort (19,181 persons), with a similar but more robust association in women than in men with NAFLD (Charatcharoenwitthaya et al.). In another article, Rim et al. suggested that early screening strategies for people with abrupt chronological changes in serum triglycerides might be useful to predict NAFLD development (Rim et al.). Indeed, a better understanding of the different factors linked to NAFLD progression will also provide insight into preventive strategies to reduce the incidence of NAFLD-associated disorders. In this regard, Abdel-Razik et al. attempted to identify risk factors for portal vein thrombosis (PVT) in NAFLD patients, finding that among all PVT-associated factors studied only an increased central obesity and an elevated leptin/adiponectin ratio in these patients are independently associated with PVT development (Abdel-Razik et al.).

Psychological disorders such as depression and anxiety are frequently present in patients with NAFLD. However, the link between mood disorders and NAFLD is still poorly understood. In this special issue, different studies have provided new insights about the association between NAFLD and mental disturbances that may have clinical implications for reducing the prevalence of comorbidities. In this sense, Choi et al. concluded that in a Korean group of study of more than 25,000 subjects, women with NAFLD show a higher tendency to suffer from depression and anxiety compared to non-NAFLD (Choi et al.). In the same way, the meta-analysis and systematic review performed by Xiao et al. suggested a strong association between depression and NAFLD. Indeed, they conclude that the worsening of NAFLD to NASH implies a higher risk of suffering depression (Xiao et al.). Moreover, it is still barely unstated how perceived social support and NAFLD development and progression alter the psychosocial profile of these patients, and which are relevant risk factors. In this special issue, Funuyet-Salas et al. demonstrated that low perceived social support, significant fibrosis, and female sex are independently associated with a higher-risk psychosocial profile in NAFLD. Therefore, a psychological intervention in NAFLD patients could be of great interest (Funuyet-Salas et al.).

The underlying mechanism for the setup and progression of NAFLD is complex and multifactorial, involving multiple parallel factors and signaling pathways. In this sense, microRNAs (miRNAs) function as critical post-transcriptional negative regulators involved not only in many biological processes but also in the development of many diseases such as NAFLD. López-Pastor et al. reviewed the latest advances in knowledge about the miRNAs involved in the development of NAFLD and related diseases, and examined how this knowledge could be used to identify new non-invasive biomarkers and new pharmacological interventions for this liver disease (López-Pastor et al.). Moreover, in an updated mini-review, Delli Bovi et al. summarized the current knowledge on the role of oxidative stress in the pathogenesis and progression of NAFLD, as well as on the preventive and therapeutic strategies available. The authors reviewed the participation of the intestinal microbiota in this process, and highlighted the use of probiotics as enhancers of antioxidant defenses, as well as focused on other antioxidant therapeutic interventions, particularly, the use of vitamin E (Delli Bovi et al.). Since several studies have reported that complement system, an innate immune system, plays an important role in the pathogenesis of NAFLD, in another review, the authors explored the role and molecular mechanism of complement component 3, a protein of the innate immune system, in NASH development as well as its implication in the diagnosis and treatment of this stage of the disease (Han and Zhang).

Likewise, clinical and preclinical studies have revealed that hypoxia may play an important role in the pathophysiology and progression of NAFLD, which have been included in a mini-review by Isaza et al. Interestingly, the article by Rey et al. described for the first time that intermittent hypoxia modulates free fatty acid (FFA) uptake by upregulating the hepatic expression of the FFA translocase CD36, and partly contributes to the NAFLD setup (Rey et al.).

The diagnosis of NAFLD is actually very difficult by non-invasive methods and usually requires a liver biopsy. For this reason, many researchers and clinicians are making strong efforts to find non-invasive and accurate methods to improve the diagnosis of this prevalent disease. Bearing this in mind, the study performed by Hokkanen et al. demonstrated that low-dose computed tomography (CT) scans could be a useful tool for liver fat quantification and NAFLD diagnosis. Their results suggest that low-dose CT is a feasible and well repeatable method but amount of liver fat contributes to repeatability. The assessment of liver fat content can be used as additional information in studies where a CT scan has been done for other medical reasons (Hokkanen et al.). In this search for non-invasive NAFLD biomarkers for diagnosis and prognosis of NAFLD, another article showed that serum leptin discriminates NAFLD, and adiponectin combined with specific lipids might be useful for the NASH stratification. Moreover, insulin-like growth factor 1 (IGF1), international normalized ratio (INR), and ferritin distinguish advanced fibrosis (Marques et al.). In another review submitted to this special issue, Garcia-Martinez et al. performed an exhaustive analysis on the literature regarding the extracellular vesicles (EV) link with hepatocytes stress which is one of the early steps leading to liver disease. Importantly, this review highlighted the potential use of EV as a new biomarker for NAFLD management (Garcia-Martinez et al.).

Due to the high prevalence of NAFLD and the absence of a specific treatment, many attempts are being done to find new therapeutic targets. In this sense, the discovery of dietary supplements is a great opportunity. Li and Zhao have analyzed the literature supporting the potential of carnitine, an amino acid-derived molecule, as a therapeutic agent for different liver diseases, particularly NAFLD. Regarding NAFLD, carnitine inhibits β-oxidation, improves mitochondrial dysfunction, and reduces insulin resistance. Therefore, carnitine supplementation may improve liver diseases including NAFLD (Li and Zhao). Yao et al. in their manuscript described the beneficial effects of myricetin, a polyphenolic flavonoid, in a murine dietary model of NASH, and even help to elucidate the underlying mechanisms: treatment with myricetin favors M2 polarity switch of liver macrophages, attenuating the activation of hepatic stellate cells and the consequent liver fibrosis (Yao et al.). Based on all this, they propose myricetin as a potential new therapeutic agent for the treatment of inflammation in NAFLD. In another article, authors explored the effect of crocetin, a bioactive ingredient of saffron, on this liver disease in a mouse model of NAFLD and revealed that crocetin ameliorates obesity-induced NAFLD by suppressing of oxidative stress and decreasing inflammation, demonstrating its therapeutic potential (Xu et al.).

In summary, this special issue will help to understand important aspects of NAFLD setup and progression. The articles published in this special issue show novel perspectives about pathogenesis and diagnosis of NAFLD as well as possible therapeutic approaches to ameliorate liver injury.

## Author Contributions

ÓE, DF, YO, JE, and AG-R wrote the manuscript. All authors critically revised the manuscript for important intellectual content.

## Funding

This work was supported by grants RTI-2018-095098-B100 from Ministerio de Ciencia, Innovación y Universidades (Spain) and PR75/18-21572 from Santander-UCM (Spain) to OE; grants CPII19/00005 and PI19/00082 from Instituto de Salud Carlos III (ISCIII, Spain) and Fondo Europeo para el Desarrollo Regional (FEDER) to JE; grants CPII19/00032, PI19/00123, and CIBERDEM from ISCIII/FEDER to AG-R.

## Conflict of Interest

The authors declare that the research was conducted in the absence of any commercial or financial relationships that could be construed as a potential conflict of interest.

## Publisher's Note

All claims expressed in this article are solely those of the authors and do not necessarily represent those of their affiliated organizations, or those of the publisher, the editors and the reviewers. Any product that may be evaluated in this article, or claim that may be made by its manufacturer, is not guaranteed or endorsed by the publisher.

